# Stage or size? The identity of anatomical and visual outcomes in stage 3 and stage 4 idiopathic macular holes after vitrectomy

**DOI:** 10.1186/s12886-023-02820-9

**Published:** 2023-03-07

**Authors:** Yanping Yu, Xida Liang, Zengyi Wang, Jing Wang, Biying Qi, Wu Liu

**Affiliations:** 1 Beijing Tongren Hospital, Beijing Tongren Eye Centre, Capital Medical University, No 1, Dongjiaominxiang, Dongcheng District, Beijing, 100730 China; 2 Beijing Ophthalmology and Visual Sciences Key Laboratory, Beijing, China

**Keywords:** Idiopathic macular hole, Minimum linear diameter, Optical coherence tomography, Stage, Vitrectomy

## Abstract

**Background:**

Several previous reports suggested that stage 4 idiopathic macular holes (IMHs) may exhibit lower rate of anatomical success and poorer functional results comparing with stage 3 IMHs, while some others showed no differences. Actually, few studies focused on comparison of prognosis between stage 3 and stage 4 IMHs. Our previous study found that IMHs of these two stages demonstrate similar preoperative characteristics, and this study aims to compare anatomical and visual outcomes of IMHs between stage 3 and stage 4, and tries to figure out the outcome-associated factors.

**Methods:**

This retrospective consecutive case series reviewed 317 eyes with IMHs of stage 3 and stage 4 from 296 patients who underwent vitrectomy with internal limiting membrane peeling. Preoperative characteristics like age, gender, and hole size, and intraoperative interventions such as combined cataract surgery were evaluated. Outcome measures included the primary closure rate (type 1), best-corrected visual acuity (BCVA), foveal retinal thickness (FRT) and prevalence of outer retinal defect (ORD) at the last visit. The pre-, intra-, and post-operative information were respectively compared between stage 3 and stage 4.

**Results:**

The preoperative characteristics and intraoperative interventions exhibited no significant differences between stages. With comparable follow-up durations (6.6 vs. 6.7 months, P = 0.79), IMHs of the two stages exhibited similar primary closure rate (91.2% vs. 91.8%, P = 0.85), BCVA (0.51 ± 0.12 vs. 0.53 ± 0.11, P = 0.78), FRT (134.8 ± 55.5 μm vs. 138.8 ± 60.7 μm, P = 0.58), and prevalence of ORD (55.1% vs. 52.6%, P = 0.39). IMHs, either < 650 μm or larger, exhibited no significant difference in outcomes between the two stages. However, smaller IMHs (< 650 μm) demonstrated higher rate of primary closure (97.6% vs. 80.8%, *P* < 0.001), better postoperative BCVA (0.58 ± 0.26 vs. 0.37 ± 0.24, *P* < 0.001), and thicker postoperative FRT (150.2 ± 54.0 vs. 104.3 ± 52.0, *P* < 0.001) comparing with larger ones regardless of stage.

**Conclusion:**

IMHs of stage 3 and stage 4 exhibited considerable identity of anatomical and visual outcomes. In large IMHs, the hole size, instead of stage, may be more important for prediction of surgical outcomes and choice of surgical techniques.

## Introduction

Idiopathic macular holes (IMHs) are defined as full-thickness dehiscence of the foveal neurosensory retina [1], which commonly leads to central vision loss and metamorphopsia. With an incidence of around 0.1–0.3% among population [2–5], IMH usually affects the elderly with a female to male ratio of 3.3 to 1 [5]. Vitrectomy together with internal limiting membrane peeling and intravitreous tamponade has been considered as an efficient treatment owing to resolution of the tangential and/or anteroposterior vitreofoveal traction.

In 1995, Gass updated his classification of IMH, considering full-thickness macular holes (FTMHs) larger than 400 μm with vitreomacular separation as stage 3, and FTMHs with complete posterior vitreous detachment as stage 4 regardless of their diameters [6]. The International Vitreomacular Traction Study Group also gave a new classification in 2013 based on optical coherence tomography (OCT) observation [7], but that of Gass’s is still widely applied nowadays.

According to our previous study, stage 3 and stage 4 IMHs exhibited similar preoperative characteristics, including duration of symptoms, preoperative visual acuity, and size of the hole depicted by minimum linear diameter, basal diameter, etc [8]. Regarding prognosis of the two stages of IMHs, several reports suggested that stage 4 IMHs seemed to exhibited lower rate of anatomical success and poorer functional results comparing with stage 3 IMHs [9–11], while others showed no differences [12]. With the current literature, few studies actually focused on this issue, and the differences between stage 3 and stage 4 IMHs still need to be fully elucidated.

The current investigation evaluates the anatomical and visual outcomes of a relatively large population, and attempts to identify whether IMHs of the two stages differ in terms of surgical prognosis.

## Materials and methods

A consecutive series of patients with stage 3 or stage 4 IMHs who underwent vitrectomy together with internal limiting membrane peeling in Beijing Tongren Hospital between July 2015 and January 2019 were reviewed. Eyes with traumatic macular holes, high myopia (axial length larger than 26.00 mm or refractive error >-6.00D), other fundus diseases like age-related macular degeneration, history of vitreous surgery or intravitreal injection, or treated with inverted internal limiting membrane flap were excluded. All the patients provided written informed consents after detailed explaining of surgery and follow-up examinations. This study adhered to the tenets of the Declaration of Helsinki and was approved by the ethical review committee of Beijing Tongren Hospital, Capital Medical University. 

Preoperative information review included a comprehensive medical history, duration of symptoms, preoperative best-corrected visual acuity (BCVA), intraocular pressure (by Full Auto Tonometer TX-F; Canon Canada, Quebec) and axial length (from IOL Master Biometry, Carl Zeiss Meditec, Jena, Germany). The anterior segment examination was performed by slit-lamp bio-microscopy, and the binocular indirect ophthalmoscope together with the fundus photography (fundus camera, TRC-50; Topcon, Tokyo, Japan) and OCT (Cirrus high-definition OCT; Carl Zeiss, Dublin, CA) was utilized for fundus examination. The diagnosis of IMH was based on detailed clinical history, comprehensive ocular examination, and intraoperative observation. Stages of macular holes were identified by OCT (status of vitreomacular adhesion) image and intraoperative observation (Weiss ring presentation) according to Gass’s classification in 1995.

Minimum linear diameter (MLD) was defined as the narrowest distance between the rims of the IMH paralleled to the retinal pigment epithelium (RPE) on a horizontal scan through the center of the hole, and basal diameter (BD) was the span of the broken ends of the neuroepithelium at the level of RPE. The main outcomes were the primary closure rate of IMH, the BCVA at the last visit, the foveal retinal thickness (FRT) at the last visit, and the prevalence of outer retinal defect (ORD) at the last visit. The closure of IMH was defined as type 1 closure [13], i.e. closed without RPE exposure in the fovea. FRT was the vertical distance between the inner surface of the retina and the roof of RPE manually measured at the lowest point of the foveal neurosensory retina. The ORD was defined as a lucency of the outer foveal retina from the external limiting membrane to the RPE. All the morphological parameters including MLD, BD, FRT and ORD were obtained by OCT with the Macular Cube 512 × 128 protocol in the horizontal direction, and was measured three times by a single observer to reach an average value.

All the patients enrolled underwent the standard 3-port pars plana vitrectomy by a single surgeon with the Constellation device (Alcon, Fort Worth, TX) by 23-gauge technique. A combined phacoemulsification and intraocular lens (CT ASPHINA 509 M; Carl Zeiss Meditec Inc, Jena, Germany) implantation was performed through a 3.0-mm incision prior to the vitrectomy when necessary. After core vitrectomy, the posterior vitreous detachment was induced if necessary and the removal of vitreous was carried out to the vitreous base. The epiretinal membrane was peeled if presented, and the internal limiting membrane was removed with a diameter of approximately 2 optic disk diameters around the macular hole without staining. Before July 2016, gas tamponade (14% hexafluoroethane or 12% octafluoropropane) was injected after fluid-air exchange; since then, sterile air was retained in vitreous cavity. The three incisions closed automatically, and the intraocular pressure was kept around 20 mmHg (gas tamponade) or 25 mmHg (air tamponade). Trans-sclera suture was only performed when there was gas or air leaking. Every patient was asked to keep strict face-down position for 1 (air tamponade) or 2 weeks (gas tamponade), and further consultation was arranged as 1 week, 1 month, 4 months, 10 months after surgery, and once a year afterwards. Postoperative pharmaceutical protocol included topical antibiotics and anti-inflammatory agents.

All data were presented as mean ± standard deviation. BCVA was converted to the logarithm of the minimum angle of resolution (Log MAR) for statistical analysis. Comparisons of categorical variables were carried out by Chi-square test or Fisher’s exact test; t test or Mann-Whitney test were conducted for continuous variables conformed to normal distribution or not respectively. Wilcoxon signed rank test was used to compare pre- and postoperative measurements. Pearson correlation and Spearman correlation were conducted to find out the association among MLD, FRT, ORD and postoperative BCVA. SPSS 22.0 (SPSS for Windows, Chicago, IL) was applied and *P* < 0.05 was considered statistically significant.

## Results

A total of 317 eyes from 296 patients (61 men and 235 women) were recruited. The average age of the patients were 65 years old (ranging from 48 to 84), and the duration of symptoms ranged from 1 to 96 months with an average of 9. IMHs of stage 3 and stage 4 showed no significant difference in proportion of duration > 9 months (69/204 vs. 39/114, *P* = 0.90). During a mean follow-up of 6.7 months (range: 1–36), three patients with stage 4 IMH were lost to follow-up with unknown closure status (one died and another two out of contact). The type 1 closure rate after first surgery was 91.4% (287/314). Among the 27 eyes with initially unclosed holes, four exhibited type 2 closure (open/flat) after surgery and remained stable during follow-up, one closed spontaneously 3 months after the initial surgery, fourteen underwent a second surgery and obtained type 1 closure, and the other eight refused the repeat operation. Eyes with gas tamponade exhibited no significant differences in the primary closure rate between stage 3 and stage 4 (97.4% vs. 95.1%, *P* = 0.61), so did eyes with air tamponade (87.5% vs. 89.9%, *P* = 0.62).

Mean BCVA at last visit of all the eyes improved significantly from 0.12 ± 0.10 to 0.51 ± 0.27 (*P* < 0.001), and no complications like retinal detachment had occurred till the last visit.

Preoperative characteristics like duration of symptoms, BCVA, and size of the holes depicted by MLD and BD exhibited no prominent differences between the two stages, which was similar to what we published in the previous report [8].

Concerning the surgical procedure, percentage of combined cataract surgery were comparable between stages (93.6% vs. 91.2%, *P* = 0.42), and rate of air tamponade were similar (62.7% vs. 61.1%, *P* = 0.77) as well.

After surgery, the mean follow-up for stage 3 and stage 4 IMHs were 6.64 and 6.69 months respectively, which exhibited no significant difference (*P* = 0.79) either. For postoperative parameters, IMHs of the two stages showed similar primary closure rate, postoperative BCVA, FRT, and presence of ORD (listed in Table 1). In either stage, FRT was negatively correlated with MLD (stage 3: *P* < 0.001, r=-0.45; stage 4: *P* = 0.001, r =-0.35) while positively correlated with postoperative BCVA (stage 3: *P* < 0.001, ρ = 0.27; stage 4: *P* = 0.001, ρ = 0.34).

IMHs with MLD < 650 μm exhibited no significant differences in the four main outcomes between stage 3 and stage 4, neither do IMHs with MLD ≥ 650 μm (shown in Table 2). However, IMHs which were smaller than 650 μm showed higher rate of primary closure (97.6% vs. 80.8%, *P* < 0.001), better postoperative BCVA (0.58 ± 0.26 vs. 0.37 ± 0.24, *P* < 0.001), and thicker postoperative FRT (150.2 ± 54.0 vs. 104.3 ± 52.0, *P* < 0.001) comparing to larger ones regardless of stage.

**Fig. 1:**
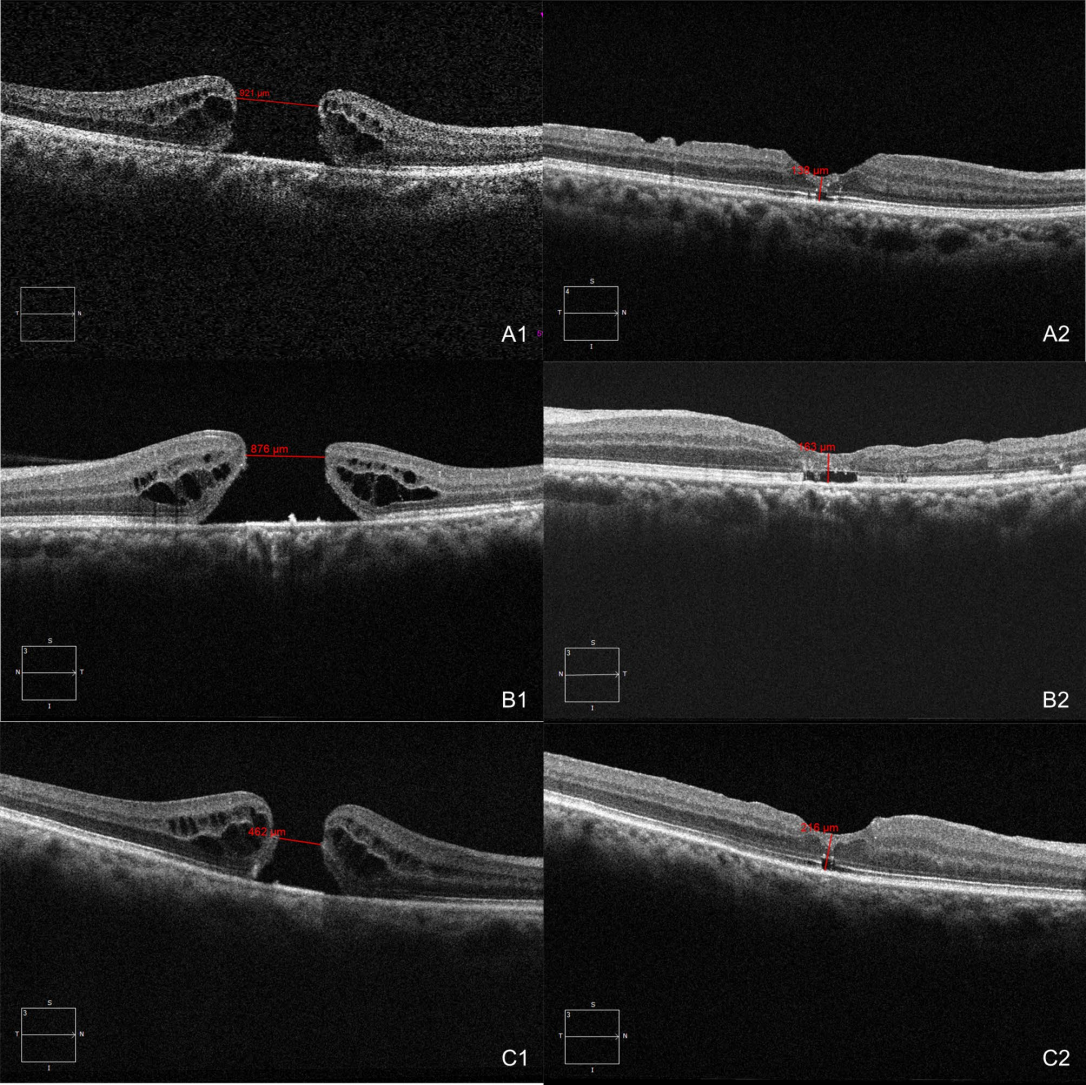
Baseline and follow-up OCT images of three representative cases. (A1, A2) Case (1) A 67-year-old woman with IMH of stage 4 (MLD = 921 μm). Ten months after surgery, she exhibited satisfactory recovery with a BCVA of 0.5 and an FRT of 138 μm. (B1, B2) Case (2) A 56-year-old man with IMH of stage 3 (MLD = 876 μm). Ten months postoperatively, he also demonstrated a BCVA of 0.5 and an FRT of 163 μm. Except for stage of the macular hole, case 1 and case 2 showed similar size and surgical outcomes. (C1, C2) Case (3) A 71-year-old man with a relatively small (MLD = 462 μm) IMH of stage (4) Seven months after surgery, he exhibited BCVA of 0.8 with an FRT of 216 μm, which recovered more quickly and better than both case 1 and case 2. OCT, optical coherence tomography; MLD, minimum linear diameter; BCVA, best-corrected visual acuity; FRT, foveal retinal thickness

Three representative cases were shown in Fig. 1. Holes of both stages achieved primary closure of IMH, and satisfactory BCVA and FRT with ORD, however, smaller IMH exhibited better outcomes.

**Table 1:** Comparison of stage 3 and stage 4 idiopathic macular holes

	Stage 3 (N = 204)	Stage 4 (N = 113)	P
Age, years	64.74 ± 5.46	64.98 ± 4.53	0.65 ^a^
Gender, M/F	37/167	29/84	0.11 ^b^
Eye, OD/OS	98/106	59/54	0.48 ^b^
Duration of symptoms, months	8.40 ± 9.31	9.96 ± 11.07	0.35 ^a^
IOP, mmHg	15.46 ± 3.21	15.42 ± 3.10	0.87 ^a^
AXL, mm	23.25 ± 0.85	23.35 ± 0.82	0.31 ^c^
Preoperative BCVA, Log MAR	1.05 ± 0.36	1.04 ± 0.36	0.81 ^a^
MLD, µm	608.2 ± 122.0	598.3 ± 157.2	0.57 ^c^
BD, µm	1158.0 ± 208.0	1128.8 ± 229.7	0.25 ^c^
Lens status, phakic/pseudophakic	201/3	110/3	0.46 ^b^
Combined PEI	93.6% (191/204)	91.2% (103/113)	0.42 ^b^
Tamponade, gas/air, percentage of air tamponade	76/128, 62.7%	44/69, 61.1%	0.77 ^b^
Primary closure rate	91.2%	91.8%	0.85 ^b^
Postoperative BCVA, Log MAR	0.38 ± 0.32	0.38 ± 0.34	0.78 ^a^
Postoperative FRT, µm	134.8 ± 55.5	138.8 ± 60.7	0.58 ^c^
Prevalence of ORD	55.1%	52.6%	0.39 ^b^
Follow-up, month	6.64 ± 7.08	6.69 ± 6.64	0.79 ^a^

a, Mann-Whitney test; b, Chi-square test; c, t-test

IMH, idiopathic macular hole; IOP, intraocular pressure; AXL, axial length; BCVA, best-corrected visual acuity; Log MAR, logarithm of the minimum angle of resolution; MLD, minimal linear diameter; BD, basal diameter; PEI, phacoemulsification and intraocular lens implantation; FRT, foveal retinal thickness; ORD, outer retinal defect.

**Table 2:** Prognostic comparisons between stages on different size of idiopathic macular holes

	MLD < 650 μm (N = 208)			MLD ≥ 650 μm (N = 106)		
	Stage 3 (N = 134)	Stage 4 (N = 74)	P	Stage 3 (N = 68)	Stage 4 (N = 38)	P
Primary closure rate	97.0%	98.6%	0.67^a^	80.9%	81.6%	0.93^b^
Postoperative BCVA, Log MAR	0.32 ± 0.32	0.27 ± 0.22	0.40^c^	0.49 ± 0.31	0.60 ± 0.42	0.27 ^c^
Postoperative FRT, µm	149.3 ± 52.8	151.7 ± 56.6	0.96 ^c^	104.2 ± 48.8	108.0 ± 60.1	0.75^d^
Prevalence of ORD	60.0%	59.7%	0.97 ^b^	53.4%	35.7%	0.12 ^b^

a, Fisher’s test; b, Chi-square test; c, Mann-Whitney test; d, t-test

IMH, idiopathic macular hole; BCVA, best-corrected visual acuity; Log MAR, logarithm of the minimum angle of resolution; FRT, foveal retinal thickness; ORD, outer retinal defect.

## Discussion

It has been suggested that stage 4 IMHs seem to be associated with less satisfactory surgical outcomes comparing to stage 3 IMHs [9, 11]. This may be explained by the histological findings that the percentage of internal limiting membrane with vitreous remnant is higher in stage 4 holes against in stage 3 holes, so that stage 4 holes may be more difficult to be sealed if the extent of internal limiting membrane peeling is not sufficient [14, 15]. However, in the report of Brockmann and colleagues’, stage 4 IMHs exhibited a closure rate without statistical significance comparing to that of stage 3, though lower in value [10]. Meanwhile, according to the comparison of the initially closed cases and unclosed cases by Hasegawa and colleagues, there was no significant difference in primary closure rate between stage 3 and stage 4 IMHs [12]. Actually, surgical outcomes include not only the closure rate, but also the visual function and other morphological parameters. Whether IMHs of the two stages differ in surgical outcomes still needs direct evidence for further elucidation, but few targeted studies have discussed about it. The present study focused on IMHs of stage 3 and stage 4 with a relatively large sample, and for the first time compared their visual function and morphological manifestations after vitrectomy. The results demonstrate that IMHs of the two stages are identical in anatomical and visual outcomes.

As is shown in our previous report [8] and confirmed in this study, IMHs of stage 3 and stage 4 exhibited no significant differences in preoperative clinical features and morphological characteristics. According to Gass’s classification applied in this study, the overwhelming majority of stage 3 and stage 4 IMHs were larger than 400 μm, so the size of the hole tended to be approximate between stages. Moreover, with posterior vitreous detachment accomplished in the fovea, the adhesion at the optic disc seems no longer to affect the evolution of the hole, so that IMHs of the two stages demonstrate identical characteristics.

Vitrectomy with internal limiting membrane peeling is considered as an efficient surgical treatment for IMHs with a primary closure rate of more than 90% according to previous reports [16]. Conventionally, gas tamponade like sulfur hexafluoride and octafluoropropane is applied. Recent years, internal limiting membrane peeling with air tamponade also exhibited satisfactory outcomes [12, 17]. In this study, gas and air tamponade were respectively applied in two time periods because gas were not available after July 2016 in mainland China. Different tamponade may affect the outcomes of surgery [18], but the two stages in this study exhibited comparable ratio of gas/air tamponade, suggesting that types of tamponade may not make a difference in comparison of the outcomes. In fact, in eyes with neither gas nor air tamponade did the primary closure rate exhibit a difference between stage 3 and stage 4. Since the age of patients and the preoperative BCVA were comparable between the two stages, it is not surprising that the rate of combined cataract surgery exhibited no difference.

Postoperatively, the primary closure rate exhibited no significant differences between the two stages. Eleven eyes with stage 3 IMHs and three eyes with stage 4 IMHs underwent a second operation and obtained final closure; five eyes with stage 3 IMHs and three eyes with stage 4 IMHs did not take a second operation. There seems to be no evident difference in the secondary closure rate between stages, but statistical analysis was not carried out considering the relatively small sample. The postoperative BCVA is considered to be associated with not only the preoperative minimum diameter [19], base diameter [19, 20], but also the primary closure [16]. Therefore, within a comparable duration of follow-up, it is understandable that the BCVA at last visit appeared identical between stage 3 and stage 4, since IMHs of the two stage exhibited comparable size and primary closure rate.

Besides closure rate and postoperative vision, the morphology of the fovea after surgery is also noteworthy. In a prospective interventional series by Apostolopoulos et al., the thickness of the fovea was manually measured and exhibited significant association with the BCVA 1 year postoperatively [21]. Takamura et al. also found the average central retinal thickness 1 month after surgery, which was computed automatically within 1 mm diameter central area, was correlated with the macular hole diameter and the BCVA 1 year after surgery [22]. Given different methods and point-in-time of the measurements, the relationship among MLD, postoperative FRT, and postoperative BCVA needs to be further discussed. In the present study, the manually measured FRT exhibited no significant difference between stages at the end of the comparable follow-up; for IMHs of either stage, FRT was indeed negatively correlated with MLD and positively correlated with the BCVA at the same point-in-time. However, whether the FRT at this moment has influence on long-term vision recovery still needs to be elucidated by further studies.

The ORD in this study, which is also referred as foveolar lucency, is a common cystic space underlying the fovea with continuous inner retina in the surgically sealed macular hole [23]. Kang et al. reviewed 96 eyes with IMH (mean MLD around 425 μm) and considered that the foveolar lucency was detected predominantly in smaller macular holes; [24] on the contrary, Grewal et al.’s report of 45 eyes (mean MLD of IMH around 227 μm) suggested that MLD ≥ 330 mm was predictive of foveolar lucency development [25]. Meanwhile, it still remains controversial whether this sign is correlated with the visual outcomes [23, 25]. In the present study, prevalence of ORD showed no significant differences between not only stage 3 and stage 4, but also smaller than 650 μm and larger. Stage 2 IMHs were not enrolled here and the mean MLD was larger than those of Kang et al’s [24] and Grewal et al’s [25], and that may be why we did not found the relationship between hole size and the prevalence of ORD. Accordingly, it could be inferred that ORD prevalence may be not correlated to stage of the hole, but to what extent ORD is influenced by hole size and whether its presence makes sense to postoperative vision still need further elucidation. That’s what we have been working on.

Considering all above, IMHs of stage 3 and stage 4 exhibited identical anatomical and visual outcomes based on comparable baseline characteristics and surgical interventions. It could be inferred that IMHs of the two stages may have an identity of the intrinsic nature in wound healing.

There are quantities of parameters provided by OCT to depict the postoperative morphology of IMH, among which the integrity of the ellipsoid zone, once referred as the IS/OS junction, is frequently applied and widely considered to be associated with the vision recovery [26, 27]. However, the definition of its abnormality varies a lot among different studies, such as defect, discontinuity, and disruption. Therefore, in the present study, two more visible anatomical parameters ——FRT and ORD, were applied to depict the fovea postoperatively.

A previous report involving 2456 IMH eyes indicated that duration > 9 months reduced the MH closure rate [28]. In this study, the proportion of duration > 9 months showed no significant difference between stage 3 and stage 4. Thus, duration of symptoms may not affect the comparison of closure rate.

This study staged IMHs according to Gass’s classification in 1995 [6] and all the IMHs enrolled were larger than 400 μm except ten of stage 4, so that the overwhelming majority here is considered as large IMHs [7]. Recently, it has been suggested that large IMHs demonstrated different success rates by a diameter around 650 μm [29, 30]. Thus, we divided all the cases into two groups by 650 μm and found in neither group did IMHs exhibit significant differences in outcomes between the two stages. However, when comparing IMHs of different sizes, those which were smaller than 650 μm exhibited significantly better outcomes than larger ones. This indicated that in large IMHs (> 400 μm), the size seems to play a more crucial role in surgical outcomes than the stage, and is a more noteworthy factor for choice of surgical intervention and prediction of prognosis.

This study of stage 3 and stage 4 IMHs has a limitation of its retrospective nature. With a relatively large sample and strict inclusion criteria, the results are considerably reliable, but further studies, especially prospective ones and randomized controlled trials are still in need. Meanwhile, the mean follow-up of about 6 months seems to be relatively short, because the visual outcomes could continue to improve in the long run [31]. However, the duration of follow-up was comparable between the two stages, so this limitation may not affect the comparison. Studies of long-term outcomes are meaningful, and that’s what we are working on.

In conclusion, this study with a relatively large sample compared stage 3 and stage 4 IMHs based on the Gass’s classification in 1995, and found IMHs of the two stages exhibited considerable identity not only in preoperative characteristics, but also in anatomical and visual outcomes after vitrectomy, internal limiting membrane and other comparable intraoperative interventions. For IMHs larger than 400 μm, the size, instead of the stage, may be more valuable for outcome prediction and decision-making in proper technique for hole closure.

## Data Availability

The datasets used and/or analysed during the current study are available from the corresponding author on reasonable request.

## Abbreviations

BCVA: Best-corrected visual acuity
BD: Basal diameter
FRT: Foveal retinal thickness
FTMH: Full-thickness macular hole
IMH: Idiopathic macular holes
Log MAR: Logarithm of the minimum angle of resolution
MLD: Minimum linear diameter
OCT: Optical coherence tomography
ORD: Outer retinal defect
RPE: Retinal pigment epithelium
